# Physical Health Comorbidity and Polypharmacy in Older Adults on Clozapine: A Quality Improvement Study

**DOI:** 10.1192/bjo.2026.11356

**Published:** 2026-06-30

**Authors:** Damodar Chari, Jessica Katanga, Neelima Yadav, Shahbaz Abdullah, Hari Subramaniam

**Affiliations:** Leicestershire Partnership NHS Trust, Leicester, United Kingdom

## Abstract

**Aims:**

This service evaluation project is aimed to assess physical health comorbidity, health monitoring, polypharmacy and health outcomes in older patients treated with clozapine.

**Methods:**

A retrospective review of electronic records was performed for the patients above 60 years who were currently prescribed clozapine. Data collected included demographic details, diagnosis, duration of treatment with clozapine, physical health status, co-prescription of medications, and physical health monitoring, along with side effects and health outcomes during the observation period.

**Results:**

50 patients between the ages of 60–89 years were identified, with the majority of patients having a diagnosis of paranoid schizophrenia. Two thirds of the cohort had more than 20 years of illness, with most having been on clozapine for more than a decade.

Patients had high physical health complexities, with more than 70% of patients having >3 physical comorbidities. Polypharmacy was prevalent, with most patients prescribed >4 medications. Physical health monitoring was complete for 50% of patients in the preceding year of assessment.

Nearly 60% of patients experienced significant side effects, the most common being constipation, hypersalivation, and hypotension. Clozapine was discontinued due to toxicity, poor concordance with monitoring, and service disengagement.

**Conclusion:**

Older patients treated with clozapine exhibited a high burden of physical comorbidity and polypharmacy despite being on high medication doses. Although known to mental health services for long duration their physical health monitoring remainedsuboptimal. These findings suggest the need for enhanced monitoring and surveillance for older patients on clozapine.

